# A spatio-temporal dataset on food flows for four West African cities

**DOI:** 10.1038/s41597-023-02163-6

**Published:** 2023-05-10

**Authors:** Hanna Karg, Edmund K. Akoto-Danso, Louis Amprako, Pay Drechsel, George Nyarko, Désiré Jean-Pascal Lompo, Stephen Ndzerem, Seydou Sidibé, Mark Hoschek, Andreas Buerkert

**Affiliations:** 1 School of Public Policy and Global Affairs, University of British Columbia, Vancouver, Canada; 2 Organic Plant Production and Agroecosystems Research in the Tropics and Subtropics, University of Kassel, Witzenhausen, Germany; 3 Physical Geography, University of Freiburg, Freiburg im Breisgau, Germany; 4 CGIAR Resilient Cities Initiative, International Water Management Institute (IWMI), Colombo, Sri Lanka; 5 University for Development Studies (UDS), Tamale, Ghana; 6 Université de Dédougou, Dédougou, Burkina Faso; 7 Institut de l’Environnement et de Recherches Agricoles (INERA), Ouagadougou, Burkina Faso; 8 Strategic Humanitarian Services (Shumas), Bamenda, Cameroon; 9 Institut d’Economie Rurale (IER), Bamako, Mali; 10 Forstliche Versuchs- und Forschungsanstalt Baden-Württemberg, Freiburg im Breisgau, Germany

**Keywords:** Agriculture, Geography, Developing world

## Abstract

Gaining insight into the food sourcing practices of cities is important to understand their resilience to climate change, economic crisis, as well as pandemics affecting food supply and security. To fill existing knowledge gaps in this area food flow data were collected in four West African cities - Bamako (Mali), Bamenda (Cameroon), Ouagadougou (Burkina Faso), and Tamale (Ghana). The data cover, depending on the city, road, rail, boat, and air traffic. Surveys were conducted for one week on average during the peak harvest, lean, and rainy seasons, resulting in a dataset of over 100,000 entries for 46 unprocessed food commodities. The data collected includes information on the key types of transportation used, quantity, source, and destination of the food flows. The data were used to delineate urban foodsheds and to identify city-specific factors constraining rural-urban linkages. The data can also be employed to inform academic and policy discussions on urban food system sustainability, to validate other datasets, and to plan humanitarian aid and food security interventions.

## Background & Summary

Food systems have been in the centre of academic and policy debates on sustainable development. Urbanisation in West Africa is a major driver of food system change, affecting agricultural land use, diets, and more^1^. The urban population in the region doubled between 2000 and 2015, whereby almost half of the population lived in cities in 2015^2^. Not only are increasing quantities of food required to feed growing urban populations, who mostly do not grow their own food and rely on food markets; growing supply chains also mean that supply lines extend further into rural areas, affecting rural livelihoods^3,4^. As a result, mid-stream segments of the food system, such as processing, storage, and transportation, have been extended and upgraded to meet rising urban demand^5–7^. Additionally, driven by a growing urban middle class, consumption patterns in cities are shifting toward more processed, fresh, and convenient food^8^, with effects on upstream production systems^9^.

Despite the importance of urban food supply, there is limited understanding of how cities in West Africa source their food, from where and in what quantities. The FAO food balance sheets^10^ provide an appraisal of food in- and outfluxes at the national scale, however, rural-urban data with a focus on cities, which also includes informal flows, is scarce.

**Fig. 1:**
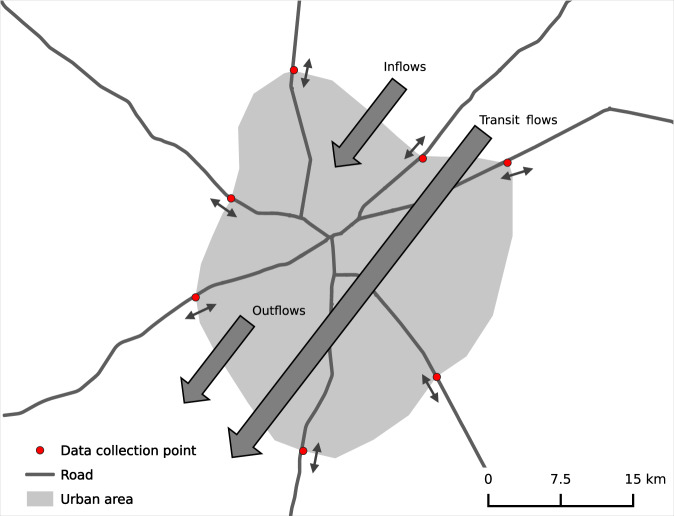
Map showing the three types of flows (inflows, outflows, and transit flows) considered in this study of the four West African cities of Bamako (Mali), Bamenda (Cameroon), Ouagadougou (Burkina Faso), and Tamale (Ghana).

Our study contributes to filling this gap by collecting geo-coded data on food flows for four West African cities over different seasons and years. The data provide insights into the quantity of food products entering and exiting the cities, their modes of transport, and their origin. The cities varying in size, accessibility, and agroecological zone were Bamako (Mali), Bamenda (Cameroon), Ouagadougou (Burkina Faso), and Tamale (Ghana). This study focused on roads as the major transport channel for food (Fig. 1). Other minor channels included the waterway on the Niger River in Bamako, the railway connection between Abidjan (Côte d’Ivoire) and Ouagadougou (Burkina Faso), and air-based transport.

**Fig. 2:**
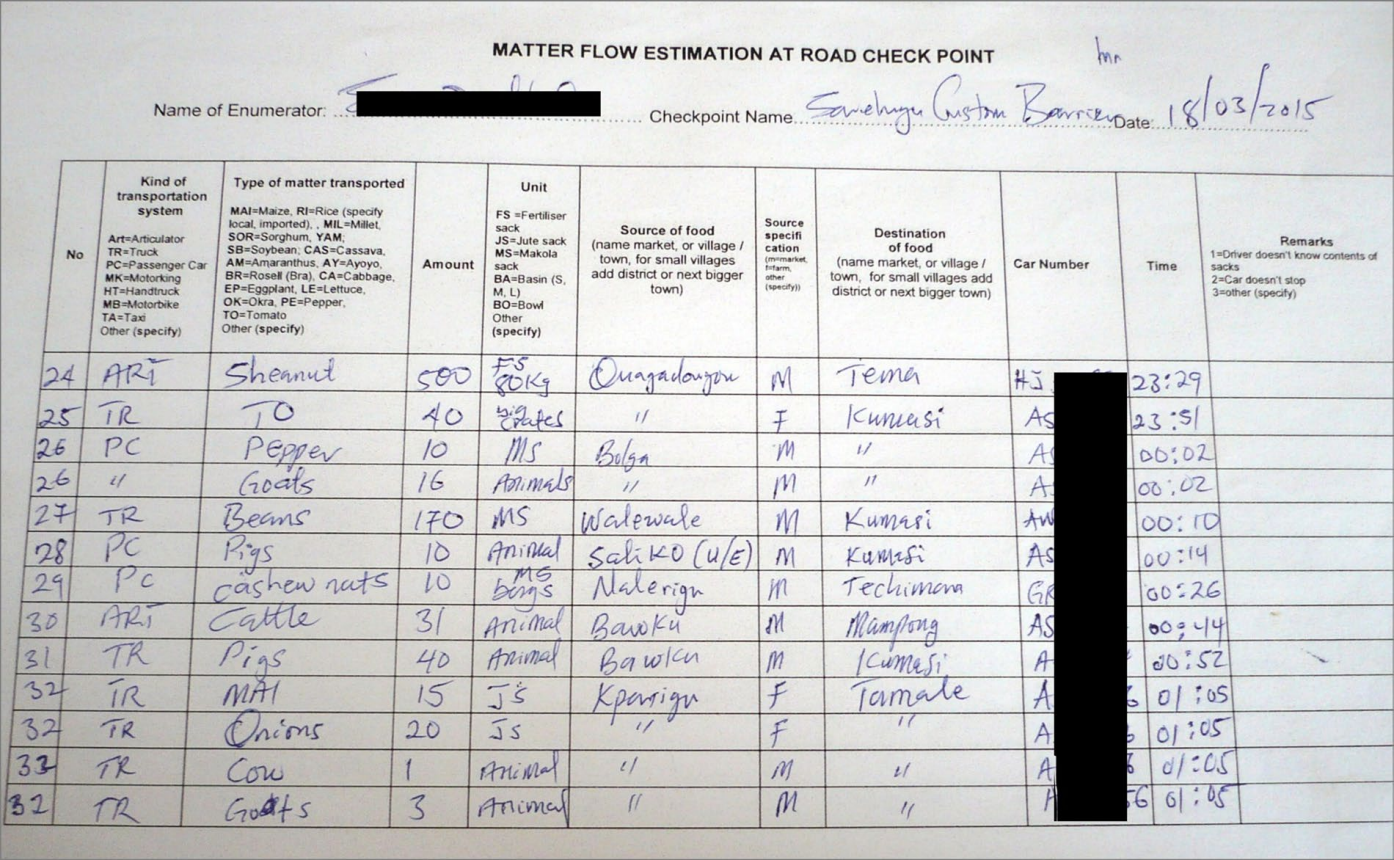
Paper form used for data collection.

Road surveys took place on all access roads to the cities where food in- and outflows were recorded on each side of the road at existing checkpoints and in collaboration with agencies operating these checkpoints. Each survey lasted one week on average, during which food movements, including the type of food, quantity and unit, geographical source, and destination, were captured (Fig. 2).

The resulting dataset contains more than 100,000 entries of flows for 46 commodities, four cities and generally three seasons^11^.

The motivation to collect the data and related research objectives were

1. to map food sources and to quantify the contribution of different spatial scales to urban food supply,
2. to examine the roles of cities in food distribution networks, and
3. to quantify virtual water and nutrients embedded in food flows.

During the past years, we used parts of the data to study foodsheds and city-regions^12^, virtual water flows^13^, livestock trade in Bamako^14^, food- and feed-based nutrient flows^15^, and the multiple roles of cities in food distribution networks^16^.

We believe, however, that the large pool of data has more potential applications, for instance in the areas of food system resilience and sustainability. Assessing food system resilience can involve specific scenarios of potential disturbances and their impact on food supply or analysing (system) properties of food networks regarding their efficiency and resilience^17^. Sustainability assessments can include calculating footprints on emissions, or land^18^, and assessing the effects of flows on distant production zones in telecoupled trade links^19^. Moreover, the data can be employed to further explore rural-urban linkages at fine spatial scales and (informal) cross-border trade flows, for which official data are scarce. They can also contribute to planning of food supply in the context of food security and for validating datasets derived from other sources, such as crop production maps.

## Methods

**Fig. 3:**
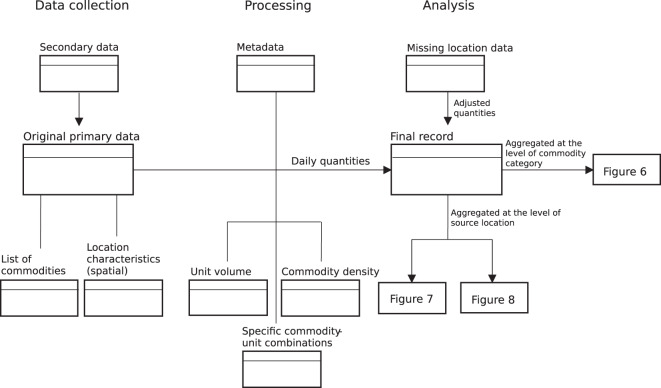
Flow diagram describing data collection, processing, and analysis of the four West African cities of Bamako (Mali), Bamenda (Cameroon), Ouagadougou (Burkina Faso), and Tamale (Ghana).

This section details the data collection and subsequent processing steps that led to the final data record^11^ (Fig. 3). We aimed to capture all food movements to and from the city by all transport types and through different channels. Prior to and during quantitative data collection, we collected qualitative data to understand food transportation and trade in their specific contexts. These included visits to urban and rural markets and primary production areas, as well as interviews with traders, transporters, farmers, and municipal authorities in charge of markets and transport. The surveys took place between 2013 and 2017 in different seasons (Table 1). The recording period was 6–8 days, depending on the length of the market week in the area, which ranged from 3–8 days. For centuries, markets have taken place periodically^20^, and adjusting the survey period to the length of the market week allowed capturing flows from all markets in the area supplying food to the city.

**Table 1:** Number of entries per survey in the four West African cities of Bamako (Mali), Bamenda (Cameroon), Ouagadougou (Burkina Faso), and Tamale (Ghana), and missing values for quantity, unit, source, and destination (in % of total counts) in the final dataset.

City	Year	Season	Total (counts)	Missing values (in % of total counts)			
				Quantity	Unit	Source	Destination
Tamale	2013	Peak (harvest)	7228	1	1	13	13
	2014	Lean (hot)	6367	2	2	7	8
	2014	Peak (harvest)	7304	2	2	10	9
	2015	Lean (hot)	6631	0	0	15	13
	2016	Rainy	4057	1	1	17	15
Ouagadougou	2014	Peak (harvest)	6771	0	0	29	28
	2014	Lean (hot)	6022	0	0	40	40
	2016	Rainy	10,336	0	0	47	47
Bamako	2015	Peak (harvest)	7609	0	0	30	30
	2016	Lean (hot)	12,378	0	1	40	40
	2017	Rainy	16,831	0	0	56	57
Bamenda	2017	Lean (hot)	9511	0	1	4	4
	2017	Rainy	12,893	0	3	11	11
TOTAL			**113,938**				

Note: The final dataset only contains selected commodities; duplicates have been removed. The number of days per survey varied and the number of data entries does not correspond to the number of trips as records are commodity-specific and one vehicle can carry several commodities.

### Data collection

#### Road survey

**Fig. 4:**
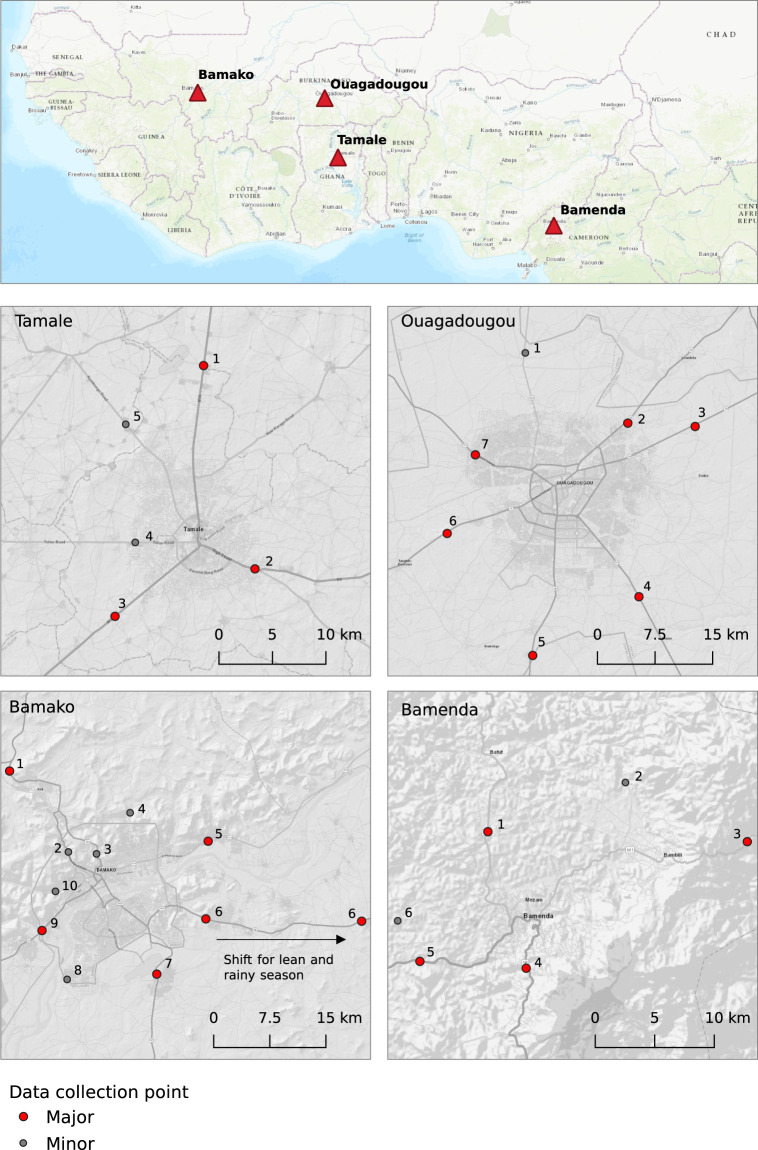
Location of major and minor data collection points along access roads to the four studied West African cities of Bamako (Mali), Bamenda (Cameroon), Ouagadougou (Burkina Faso), and Tamale (Ghana) (Map sources: ESRI, 2023^24,25^. Note: North of Bamako, data were collected at three checkpoints: two minor checkpoints close to the city boundary (#2, #3), where peri-urban food sources could be captured, and one major checkpoint at the main checkpoint in Kati (#1), where large, long-distance trucks stop. More detailed information on the checkpoints can be found in Supplementary Table 1^21^.

**Fig. 5:**
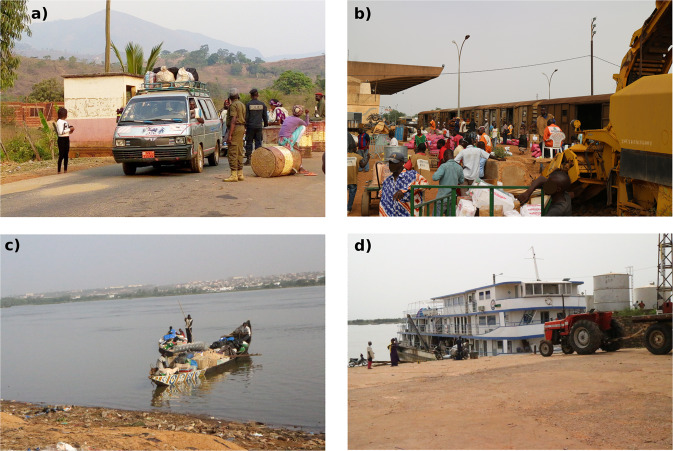
(**a**–**d**) Transport channels for food via a) road (in Bamenda; source: Prisly Dzesinyuy), b) rail in Ouagadougou, and via the Niger River in Bamako (from upstream [5c] and downstream sources recorded in Koulikoro near Bamako, Mali [5d]).

Roads are the major channel for food transportation in Bamako and Ouagadougou and the only channel for food transportation in Tamale and Bamenda. We covered access roads for 24 hours (major road), and 12 hours per day (minor road) during the survey period at existing checkpoints, operated by different agencies controlling the traffic (Fig. 4). In Bamako, checkpoints were operated by a number of agencies, including phytosanitary and forestry services. In Bamenda and Tamale, security forces were in charge of checkpoints, and in Ouagadougou, tollgates served as data collection points. We acquired formal authorization to work at these checkpoints and built a relationship with staff on the ground, whose support was crucial for successful data collection. Enumerators, who had been thoroughly trained and supervised, recorded flow data on both sides of the road (Fig. 5a). When vehicles stopped, enumerators talked to the driver or the driver’s assistant and recorded the basic information, including the type of product, quantity, and unit, as well as geographical source and destination of flows (Fig. 2). In cases where enumerators were unable to talk to the driver (if a vehicle did not stop or the driver was reluctant to share information), the transported quantity was estimated, but source and destination locations could not be determined. This was the case for an average of 8% in Bamenda, 12% in Tamale, 38% in Ouagadougou, and 42% in Bamako (Table 1).

We recorded unprocessed food products, livestock, firewood and charcoal, animal feed, and slightly processed products, such as flour. The published data is a subset of the original list of commodities and contains the most common 46 unprocessed and slightly processed crops (Table 2), fish and livestock (Table 3). Not included in the dataset are leafy vegetables commonly produced and consumed in urban and peri-urban areas of the cities and therefore rarely crossing the checkpoints^16^.

**Table 2:** List of unprocessed and slightly processed crops in the dataset of the four West African cities of Bamako (Mali), Bamenda (Cameroon), Ouagadougou (Burkina Faso), and Tamale (Ghana).

Commodity	Category	Botanical name
Maize	Cereal	*Zea mays*
Millet	Cereal	*Pennisetum americanum*
Rice	Cereal	*Oryza sativa*
Sorghum	Cereal	*Sorghum bicolor*
Wheat	Cereal	*Triticum spp*.
Wheat flour	Cereal	*Triticum spp*.
Banana	Fruit	*Musa paradisiaca*
Mango	Fruit	*Mangifera indica*
Orange	Fruit	*Citrus sinensis*
Papaya	Fruit	*Carica papaya*
Pineapple	Fruit	*Ananas comosus*
Watermelon	Fruit	*Citrullus lanatus*
Bambara bean	Legume	*Vigna subterranean*
Bean	Legume	*Phaseolus vulgaris*
Cowpea	Legume	*Vigna unguiculata*
Green bean	Legume	*Phaseolus vigna spp*.
Groundnut	Legume	*Arachis hypogaea*
Soybean	Legume	*Glycine max*
Cassava	Roots, tubers, and plantains	*Manihot esculenta*
Cocoyam	Roots, tubers, and plantains	*Colocasia spp.; Xanthosoma spp*.
Gari	Roots, tubers, and plantains	*Manihot esculenta*
Ginger	Roots, tubers, and plantains	*Zingiber officinalis*
Plantain	Roots, tubers, and plantains	*Musa paradisiaca*
Potato	Roots, tubers, and plantains	*Solamum tuberosum*
Sweet potato	Roots, tubers, and plantains	*Ipomoea batatas*
Yam	Roots, tubers, and plantains	*Dioscorea spp*.
Avocado	Vegetable	*Persea americona*
Cabbage	Vegetable	*Brassica oleracea capitata*
Carrot	Vegetable	*Daucus carota*
Chilli pepper	Vegetable	*Capsicum spp*.
Cucumber	Vegetable	*Cucumis sativus*
Eggplant	Vegetable	*Solanum melongena*
Leek	Vegetable	*Allium ampeloprasum*
Okra	Vegetable	*Abelmoschus esculentus*
Onion	Vegetable	*Allium cepa*
Spring onion	Vegetable	*Allium fistulosum*
Sweet pepper	Vegetable	*Capsicum annuum*
Tomato	Vegetable	*Lycopersicum esculentum*
Zucchini	Vegetable	*Cucurbita pepo*

**Table 3:** List of animal-based commodities in the dataset of the four West African cities of Bamako (Mali), Bamenda (Cameroon), Ouagadougou (Burkina Faso), and Tamale (Ghana).

Commodity	Category
Cattle	Livestock
Chicken	Livestock
Goat	Livestock
Guinea fowl	Livestock
Pig	Livestock
Sheep	Livestock
Fish	Fish

#### Other transport channels

In the capital cities, additional transport channels were available, but at small scale or only for few selected products. As data collection showed, food imports and exports *via* aerial transport were limited to imported high-value and mainly processed food items and exported high-value fresh products. Monthly data on export goods *via* airplane were provided to the authors by courtesy of *Air France* at the International Airport in Ouagadougou, the main airline for the export of fresh produce. Data were from January 2013 to June 2014 and consequently converted into daily quantities (May 2014, August 2013, December 2013 corresponded to lean, rainy, and peak season, respectively).

Both capital cities Bamako and Ouagadougou are connected to coastal countries *via* railway. While the railway system between Dakar (Senegal) and Bamako was not fully operational, the train connecting Abidjan (Côte d’Ivoire) with Ouagadougou operated on a regular basis (Fig. 5b), transporting imported goods, such as rice (*Oryza sativa* L.) and wheat (*Triticum aestivum* L.). Data on these goods were obtained from the railway company in Abidjan *Sitarail*. These data contained monthly flows from May 2014 to April 2015, which were converted into daily quantities (inflows in May 2014, August 2014, and December 2014 corresponded to the lean, rainy, and peak season, respectively). To account for possible off-loadings in towns between Abidjan and Ouagadougou, quantities were split according to the size of major intermediate towns, which were Bouaké in Côte d’Ivoire, and Banfora and Bobo-Dioulasso in Burkina Faso, resulting in 63.5% of the total load reaching Ouagadougou. Both rail and air freight were added to the data records, with the transport modes specified as ‘Rail’ and ‘Plane’. The data on rail and air freight were not available online and made available upon request, which involved submitting a request letter and following up in person.

Bamako is located on the banks of the river Niger, which connects the city upstream with neighbouring Guinea and downstream with northern Mali. We accounted for inflows from Guinea by small fisher boats during the peak season when the water level was high enough (Fig. 5c). However, inflows of unprocessed or only slightly processed food products were limited. In addition, we recorded incoming and outgoing goods from the port of Koulikoro, 50 km downstream from Bamako, from where boats access Ségou, Mopti, Tombouctou, and Gao (Fig. 5d).

#### Data duplicates and gaps

The study cities differed in terms of food supply and transport channels, and we had to take these differences into account and adjust data collection. This resulted in minor data gaps and duplicates. Duplicates were detected using a combination of date, time, types of products and vehicle, as well as personal details such as the number of the vehicle’s license plate, and were removed before analysis. Other adjustments are listed below:

- Bamako: Data were additionally collected at minor checkpoints to account for peri-urban food sources where major checkpoints were located further away from the city boundary (#1 and #6 in Fig. 4).
- Tamale: One road without a checkpoint was not covered (Fig. 4). To compensate for this gap, in- and outflows were recorded at the main urban wholesale market. A data gap remained when vehicles travelling along this road targeted other destinations in the city, such as warehouses. Data collected at warehouses in Tamale showed that cereal flows targeting warehouses in the city can amount to up to 30% of total recorded inflows^15^. The Tamale-Bolgatanga Road was not covered during night in the 2013 peak season survey (#1, Fig. 4). While the gap for in- and outflows was compensated by data collection at the inner-urban wholesale market, transit flows were only recorded once on one of the other road checkpoints. This must be considered when calculating transit flows using the Tamale 2013 data^21^. We also recorded goods at nearby rural markets destined for Tamale to account for diverse items from different traders, which were transported by truck and difficult to account for on the road. Both the inner-urban market and village markets were not covered during the rainy season 2016.
- Bamenda: An adjustment similar to the one in Tamale was made in Bamenda, where in- and outflows were additionally recorded at bus stations (called ‘agencies’) during loading and off-loading. Food was often transported on intercity buses, in small unit quantities, and by different passengers. Capturing these diverse products on the road would have been impossible.
- Ouagadougou: A missing permission to work at the checkpoint on the road leading to Loumbila (#2, Fig. 4) in the lean season 2014 resulted in data gaps on source and destination locations, where product types and quantities were estimated visually. Location information was estimated based on expert knowledge and visits to major production areas in the hinterland. This way, half of the records were assigned to source and destination locations^21^, representing 0.9% of total inflows in this season and, depending on the product, up to 57% of product-specific inflows (Zucchini [*Cucurbita pepo*], cattle: 45%, eggplant [*Solanum melongena*]: 29%).

### Data processing

Using an interface, data were entered online into a PostgreSQL database with a PostGIS extension, which allows handling spatial data. This way, geographical sources and destinations of food flows, for which only names were captured during data collection, could be linked to geocoded locations in a related spatial table created from a range of data sources, including OpenStreetMap data^21^. Food quantities (in fresh weight) were calculated based on unit volumes and product densities. Units transporting food, including baskets, sacks, and vehicles, as well as food density were measured *in situ* or secondary sources were used^21^. Where goods were transported in articulated trucks and the exact number of smaller units, such as the number of sacks, was not available, we used the official maximum gross weight for Ghana (which was slightly higher than in Burkina Faso) to estimate the quantity, and assumed a flat, i.e., the least heavy, unloaded trailer for the resulting net weight to account for overloading. According to informants in Ghana’s transport sector, overloading is common and not reflected in official papers, such as in waybills required for cross-border trade. For the quantification of (smaller) truck loads, we relied on the volume of the trailer and product density. Products with different processing levels such as dry and fresh fish, were quantified separately and merged later. Apart from assigning location names to geocoded locations and calibrating product quantities, data cleaning involved standardizing terms and removing duplicates. Metadata, such as number of days, daytime, season, population, were added to the dataset, and eventually daily quantities per data row calculated.

### Limitations

We aimed at a full coverage of all in- and outflows, however, the survey may have missed flows, for instance, in case of livestock herded into cities and bypassing the main roads. Thus, the flow quantities should be considered as conservative.

The limited survey period of 6–8 days does not allow the computing of the net inflow of goods to the city because products, such as dry grains but also livestock, are commonly stored or kept in the city for later export. This was the case for maize (*Zea mays* L.) in Tamale causing a negative balance when maize previously stored in urban warehouse was released, reinforced by the missed food flows targeting urban warehouses on one road.

Political instability in the region affected data collection in some cities. For instance, the peak season survey in Ouagadougou, which was originally planned for October 2014, had to be postponed to December 2014 due to the revolution in Burkina Faso. In Bamenda, the emerging political crisis prevented a third survey during the peak season. The lean and rainy season surveys in Bamenda took place during a time when general strikes, which were part of a wider protest against governmental authorities, were common and put the traffic to a stop on some days. Therefore, the survey could only take place on days without strikes, which may have led to an overestimation of daily flows on those days. In addition, challenges with security forces at selected checkpoints led to 6 out of 64 nights not covered during the rainy survey.

For intra-regional flows, the exact source and destination locations were often not known, resulting in a lower level of detail of geographic locations, thus, often only the source or destination country was available.

## Data Records

The dataset “Food_flow_data_v1” is available as .csv file via the Zenodo reference number 6423382^11^. All other files, including supplementary information, code, the PostgreSQL database structure, and auxiliary tables, are available on GitHub (https://github.com/HannaKarg/food-flows)^21^. The final dataset reflects the primary raw data entries without duplicates and with additional information on standardised quantity and geography as described in the Method section. Each row represents a product-specific flow, including quantity and geographical source and destination for the four West African cities of Bamako, Bamenda, Ouagadougou, and Tamale (Table 4). It is important to note that rows do not necessarily represent a trip as one vehicle can carry multiple items.

**Table 4:** Structure of the food flow dataset^11^.

Column name(s)	Data type	Description
id	integer	A consecutive, unique number per row
original_id	integer	The id of the original unprocessed data (Don’t use. These are city-specific and therefore do not represent a unique identifier of the data.)
data_collection_name	char	Name of the checkpoint where data were collected (for more information refer to Supplementary Table 1^21^)
direction	char	Describes entry (‘in’) or exit (‘out’). Note that transit flows are included.
means_of_transport	char	Means of transportation
commodity_name	char	Name of commodity (detailed)
commodity_name_gen	char	Name of commodity summarised (e.g., frozen, dry, smoked fish → ‘fish’)
commodity_category	char	Food group
quantity	numeric	Number of units
unit	char	Units can refer to pieces, different types of packaging (e.g., sacks), and vehicles.
source_name/destination_name	char	Name of source location
source_id/destination_id	integer	Location id referring to the original locations table. Note that locations tables are city-specific, i.e., this is not a unique identifier.
source_within_urban_boundary/destination_within_urban_boundary	char	Specifies whether source/destination location is within the urban boundary of the focal city
source_specification	char	Type of source, e.g., farm, warehouse (implemented with the peak season survey in 2014)
source_geometry/destination_geometry	WKT	Point geometry of the source/destination location
distance_to_source/distance_to_destination	double	Distance between focal city (central market) and source/destination location (km); geographical coordinates transformed in UTM (Tamale, Ouagadougou: Zone: 30 N [EPSG:32630]; Bamenda: Zone: 32 N [EPSG:32632]; Bamako: Zone: 29 N [EPSG:32629])
date	date	Date of data collection
time	time	Time of data collection
season	char	Season of data collection, thus peak, lean ( = hot), or rainy season
year	integer	Year
no_days	integer	The number of days data were collected (checkpoint-, season,- and year-specific survey)
daytime	char	Day (6am to 6 pm) or night (6 pm to 6am); ‘na’ in the case of secondary data
unit_quantity	double	Fresh weight (kg) of one unit
total_quantity	double	Unit quantity multiplied with quantity (kg)
daily_quantity	double	Total quantity divided by number of days (kg)
percent_missing_quantity	double	Share of season- and product-specific flows without location (source, destination) information (in %)
daily_quantity_adjusted_for_missing_locations	double	Daily quantity adjusted by percent_missing_quantity
population	Integer	Population of focal city^2^
city	Char	Name of focal city

It is important to note that the direction only indicates whether a vehicle was recorded when entering or exiting the city. It does not reveal whether for an incoming vehicle the focal city is the final destination or whether a vehicle passes through the city for another destination. Therefore, food flows must be classified according to different flow types prior to data analysis, depending on the application:

1. Inflows (source_within_urban_boundary = ‘no’, destination_within_urban_boundary = ‘yes’)
2. Outflows (source_within_urban_boundary = ‘yes’, destination_within_urban_boundary = ‘no’)
3. Transit flows (source_within_urban_boundary = ‘no’, destination_within_urban_boundary = ‘no’)

This classification depends on the geographical information for source and destination. Given the missing location information for parts of the dataset (Table 1), quantities should therefore be adjusted before aggregating flows. We did this by adding the missing percentage to flows with existing geographical information, which yielded the field ‘daily_quantity_adjusted_for_missing_locations’.

**Fig. 6:**
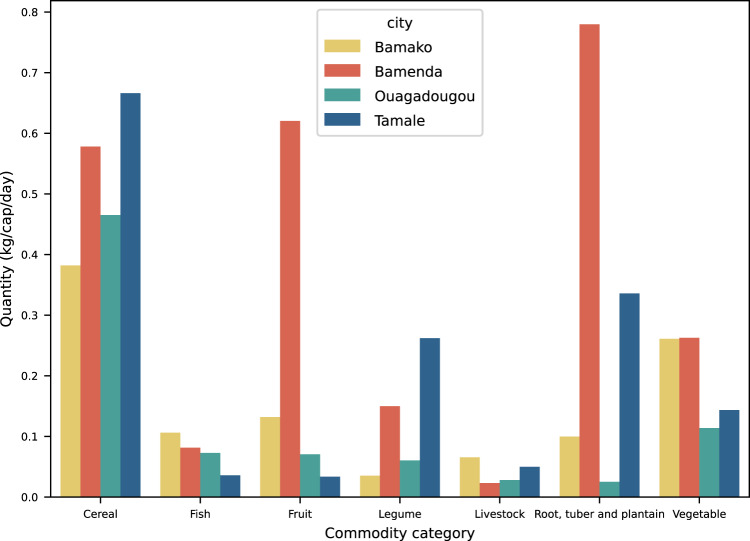
Daily per capita inflows (in kg/cap/day) per food group entering the West African cities of Bamako (Mali), Bamenda (Cameroon), Ouagadougou (Burkina Faso), and Tamale (Ghana) during the lean (hot) season.

Food flow data can be aggregated at different levels such as aggregated inflows at the level of city, food group, and season (Fig. 6) to compare food supply in cities. City-specific differences can be related to different agroecological zones of the cities. For instance, higher root, tuber, and plantain inflows in Bamenda and Tamale reflect the suitable production conditions in the area compared with the Sahelian cities Bamako and Ouagadougou^16^. One must also note that inflows do not necessarily reflect consumption because food losses, inner-urban storage, and outflows, which can be substantial in the case of storable products, have not been considered.

Inflows can also be aggregated at the level of different spatial units of origin, for example at the district, national, intra-regional, or international level. Here it is important to note that this dataset only covers the immediate upstream source and downstream destination and not the entire supply chain from farm to fork. Therefore, one must consider flows from potential hubs, such as ports or border towns, which are particularly relevant for intra-regional and international flows. Another issue concerning intra-regional flows is the already mentioned low level of detail of geographic locations, where often only the source or destination country but not the exact place within the country was known. One way to deal with these challenges is the use of secondary data to estimate the share of domestic (national) production *versus* imports (see FAO food balance sheets in^16^)

**Fig. 7:**
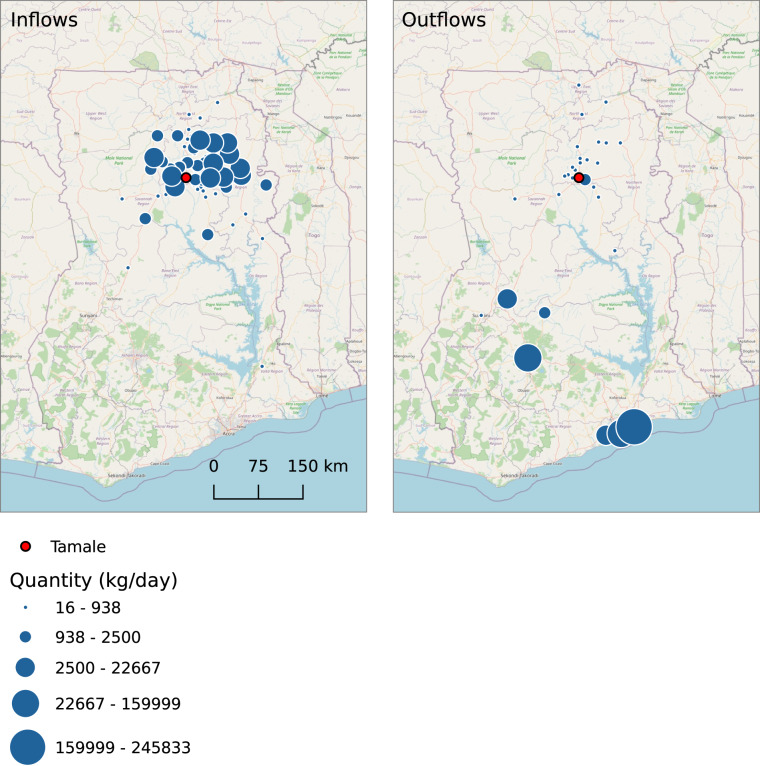
In- and outflows in kg/day of maize in the peak season in Tamale (Ghana). Note: Quantities were not adjusted for missing locations.

Plotting sources and destinations of flows on a map, with the symbol size reflecting quantities by geographic location, requires aggregation at the level of source/destination. Therefore, product(s), season, and type of flows must be extracted prior to aggregation. Figure 7 shows maize inflows and outflows (in kg/day) for Tamale during the peak season. The number of geographical sources and destinations as well as the quantities reflect Tamale’s position as aggregation centre for maize, where the city aggregated from many rural producers and small town markets in rather small quantities and sent the produce in large quantities to the major urban centres in the central and southern parts of the country^16^.

**Fig. 8:**
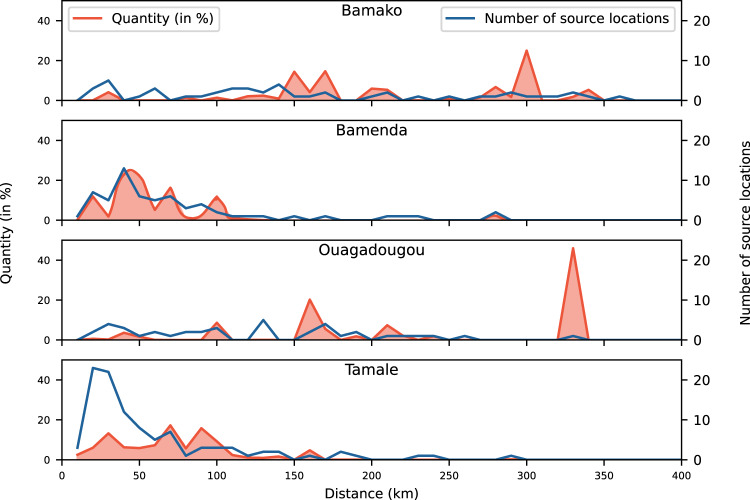
Relative inflows of maize (in %) and number of maize source locations along a distance gradient aggregated at 10 km intervals in the four West African cities of Bamako (Mali), Bamenda (Cameroon), Ouagadougou (Burkina Faso), and Tamale (Ghana).

Instead of adjusting absolute quantities by taking missing location data into account, relative quantities (in %) can be used, for instance, to describe flow characteristics along a distance gradient (Fig. 8). Using the example of maize, Fig. 8 shows how the cities relied on different distances for their maize supply. While Tamale and Bamenda sourced most maize from within a 100 km radius, Bamako and Ouagadougou obtained their maize supplies from diverse, more distant, sources (Fig. 8). Tamale and Bamenda sourced maize from many source locations, while the number of source locations for Ouagadougou and Bamako were equally low across the distance gradient, indicating more concentrated supply chains. For instance, Ouagadougou’s major supplier for maize was Bobo-Dioulasso, the second largest city in Burkina Faso, which contributed more than 40% of total maize supplies. The large number of source locations within the 50 km radius in Tamale, compared to the quantity supplied by this zone, suggests a large proportion of rural producers supplying small quantities each.

## Technical Validation

Quality controls were implemented from the onset of the study. The selection and training of enumerators involved practical tests both in the classroom and in the field. For instance, a test run was conducted prior to the official survey where enumerators collected data on the ground for one day. Unannounced controls and parallel recording of data took place on the test day and during the survey. Thorough checks were conducted on all survey books after each survey to address any open issues. Data entry was done using an entry mask with predefined data formats, minimising the risk of formal errors. The original data was digitised and compared with entered data during data entry and data cleaning.

As there is a lack of existing data on food flows, direct comparison to other datasets is not possible. However, we present a comparison of data collected across different years and a comparison of our data with secondary food supply data from FAOSTAT^22^ (Supplementary Table 2^21^).

**Fig. 9:**
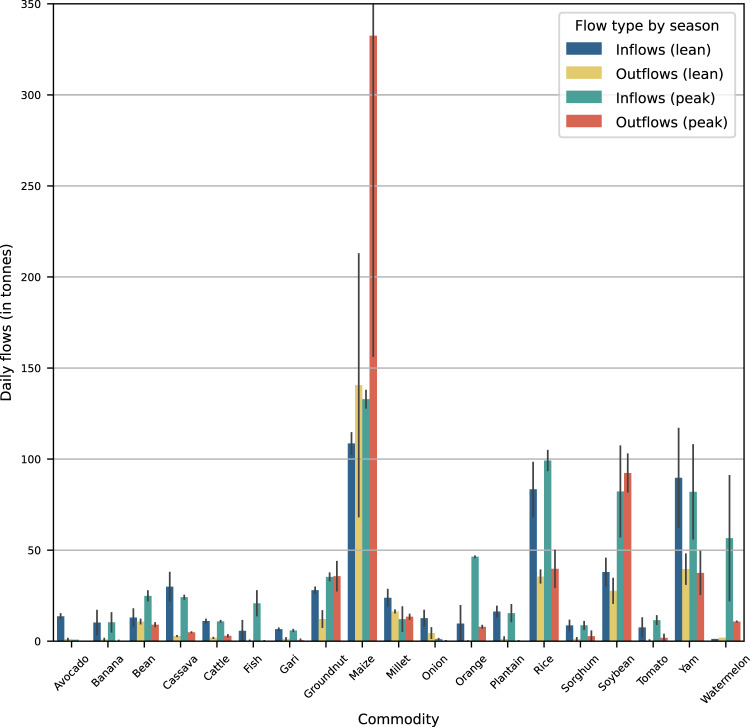
Average daily seasonal flows (in tonnes) in Tamale (Ghana) for major products over two consecutive years (peak season 2013, lean season 2014, peak season 2014, lean season 2015). Error bars indicate the minimum and maximum value.

In Tamale, we collected data for two consecutive years covering two seasons. For half of the products, the annual variation lies within 50% (compared to the respective previous year; Fig. 9). Maize outflows showed an exceptionally large variation in the peak season (224%). This is likely related to two limitations of the study (see Methods), one being the short survey period and the associated challenge to capture inner-urban storage, which may result in a negative net balance when previously stored goods were released during the survey period. The second limitation relates to the data gap for flows travelling along the road, which was not covered, and destined for inner-urban warehouses. Therefore, inflows of maize were likely higher. In other cases, it is more challenging to distinguish between sampling errors and annual differences ranging from climatic variation and harvest periods to individual marketing decisions. But given the relatively low variation between different years, we reckon that the rather short survey period captures seasonal in- and outflows well.

**Fig. 10:**
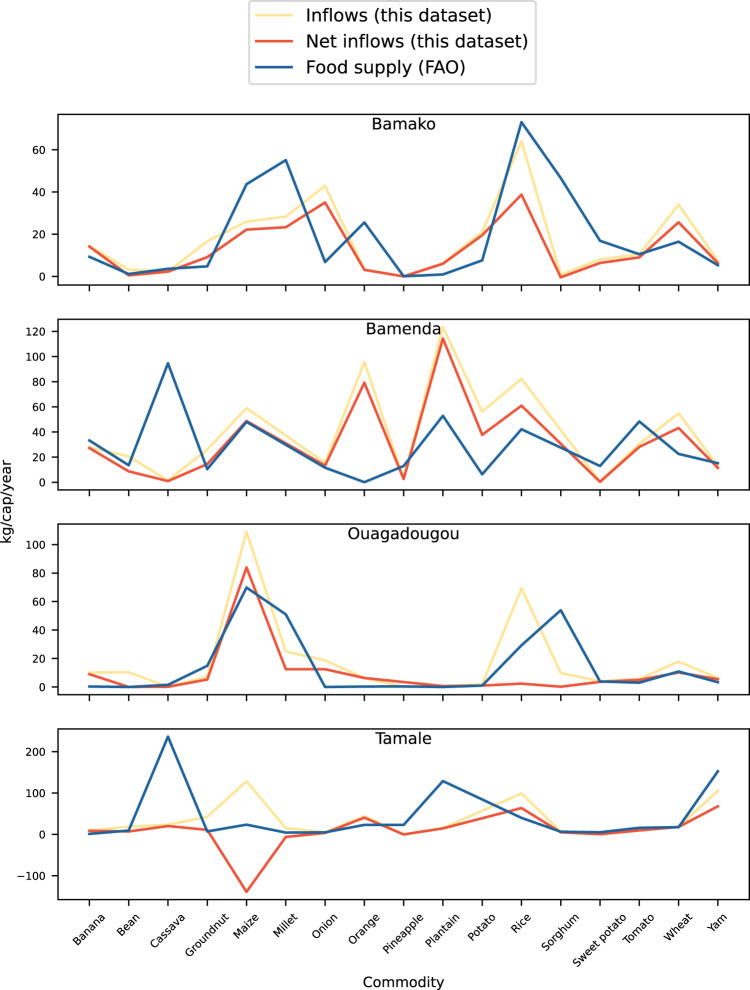
Comparison of net inflows (inflows minus outflows) with FAO food supply (in kg/cap/year) for the four studied West African cities of Bamako (Mali), Bamenda (Cameroon), Ouagadougou (Burkina Faso), and Tamale (Ghana). Annual data were derived from averaging seasonal flows.

We also compared net inflows (inflows minus outflows) with national data on food supply from FAOSTAT for the years 2014 (Tamale, Ouagadougou), 2016 (Bamako), and 2017 (Bamenda)(Supplementary Table 2^21^). The variation between the two datasets ranges from 1% (maize in Bamenda, Cameroon) to more than 26,000% (Sorghum in Ouagadougou, Burkina Faso; Fig. 10). 25 out of 64 products have a variation of less than 50%, and for almost half of the products (n = 31) FAO data show smaller values, which means that there is no overall systematic under- or overestimation.

However, there are several caveats to consider when comparing the two datasets, such as the fact that FAO data are national-level and do not take sub-national differences into account. For instance, Tamale in northern Ghana relies less on plantain and cassava than the central and southern part of the country, one possible explanation for the disparity between our data and the FAO data. It also means that the FAO data does not distinguish between rural and urban food supply, which can differ. For instance, millet and sorghum are considered rural crops in the Sahel^23^, one likely reason for the lower quantities of these crops for Ouagadougou and Bamako in our dataset. Additionally, using the difference between inflows and outflows is problematic due to the short survey period and the lack of consideration of storage and losses (s. maize for Tamale). Eventually, the extrapolation of three (two in the case of Bamenda) seasons to the entire year will certainly miss out production periods.

## Usage Notes

The Jupyter Notebook provides ways to reuse the data^21^. For instance, it offers approaches for different levels of aggregation and spatial data handling informing Figs. 6–8. It also demonstrates the extraction of transit flows and shows the magnitude of flows through the other two transport channels ‘Rail’ and ‘Plane’ in Ouagadougou. The Jupyter Notebook was written in Python. Python (3.9) and relevant packages (pyproj, scipy, matplotlib, pandas, shapely, numpy, seaborn, gdal, geopandas, jupyter, nbconvert) were installed in a Conda environment. A kernel spec (ipykernel) was created manually in this Conda environment and accessed by Jupyter Notebook from the base environment.

## Data Availability

Initially, the data were organised in a relational PostgreSQL database with separated tables for metadata, calibration lists, and spatial characteristics. The final dataset was generated in pgAdmin 4 using PostgreSQL 12 including all relevant information from these previously related tables. This included the following steps: 1. Metadata and spatial characteristics of locations joined to original data, secondary data for Ouagadougou added to the dataset, missing information on Loumbila checkpoint added (lean season 2014); duplicates removed. 2. Unit volume, product density, and specific product-unit combinations joined to the dataset. 3. Selected products extracted and livestock quantities adjusted (from number of animals to kg). 4. Missing quantity computed and adjusted quantities calculated. 5. Distance calculated and geometry added. The database structure as well as the auxiliary tables are available on GitHub^21^, however, they are not needed for reusing the data.
